# The adaptability of the ion-binding site by the Ag(I)/Cu(I) periplasmic chaperone SilF

**DOI:** 10.1016/j.jbc.2023.105331

**Published:** 2023-10-14

**Authors:** Ryan M. Lithgo, Marko Hanževački, Gemma Harris, Jos J.A.G. Kamps, Ellie Holden, Tiberiu-Marius Gianga, Justin L.P. Benesch, Christof M. Jäger, Anna K. Croft, Rohannah Hussain, Jon L. Hobman, Allen M. Orville, Andrew Quigley, Stephen B. Carr, David J. Scott

**Affiliations:** 1 School of Biosciences, Sutton Bonington Campus, University of Nottingham, Leicestershire, United Kingdom; 2 Membrane Protein Laboratory, Diamond Light Source, Rutherford Appleton Laboratory, Didcot, Oxfordshire, United Kingdom; 3 Diamond Light Source, Diamond House, Rutherford Appleton Laboratories, Didcot, Oxfordshire, United Kingdom; 4 Research Complex at Harwell, Rutherford Appleton Laboratory, Didcot, Oxfordshire, United Kingdom; 5 Department of Chemical and Environmental Engineering, University of Nottingham, University Park, Nottingham, United Kingdom; 6 Department of Chemistry, University of Oxford, Oxford, Oxfordshire, United Kingdom; 7 Department of Data Science and Modelling, Pharmaceutical Sciences, R&D, AstraZeneca Gothenburg, Mölndal, Sweden; 8 Department of Chemical Engineering, University of Loughborough, Loughborough, United Kingdom

**Keywords:** silver ion binding, metal ion chaperone, thermodynamic, QM-MM, structural biology

## Abstract

The periplasmic chaperone SilF has been identified as part of an Ag(I) detoxification system in Gram-negative bacteria. Sil proteins also bind Cu(I) but with reported weaker affinity, therefore leading to the designation of a specific detoxification system for Ag(I). Using isothermal titration calorimetry, we show that binding of both ions is not only tighter than previously thought but of very similar affinities. We investigated the structural origins of ion binding using molecular dynamics and QM/MM simulations underpinned by structural and biophysical experiments. The results of this analysis showed that the binding site adapts to accommodate either ion, with key interactions with the solvent in the case of Cu(I). The implications of this are that Gram-negative bacteria do not appear to have evolved a specific Ag(I) efflux system but take advantage of the existing Cu(I) detoxification system. Therefore, there are consequences for how we define a particular metal resistance mechanism and understand its evolution in the environment.

Silver compounds are effective antimicrobials that are highly toxic to many Gram-negative bacteria including *Escherichia coli*. Silver is nontoxic to humans and other higher eukaryotes, except if ingested in very large quantities (1, 2) unlike other bactericidal metal ions such as mercury. As such, silver compounds can be found within the linings of bandages and as additives in creams, both of which are used in hospital burn wards and as linings for medical equipment such as catheters (2, 3). Silver compounds have been used as an antimicrobial in a wide variety of household and personal products such as washing machine interiors, deodorants, and some items of clothing (4, 5, 6, 7).

With such a wide-spread use of an unlicensed antimicrobial and its unavoidable release into the environment, there has been an inevitable emergence of silver-resistant bacteria. The first cases, reported in a US hospital burns ward in the 1960s, were of resistant *Salmonella enterica* (8), but silver resistance is being reported across a wide range of Gram-negative bacteria. The archetype silver resistance genes reside on a 383 kb plasmid, pMG101 (9, 10). Studies of *E. coli* containing the plasmid pMG101 showed that the bacteria were able to survive and grow in the presence of 6× the normal lethal dosage of Ag(I) (10, 11). Monovalent silver ions, Ag(I), are the active element, rather than metallic silver itself (12, 13, 14). Therefore, the structural requirements for recognition of Ag(I) *versus* Cu(I) are of great interest to understand not only silver metal ion resistance but also how proteins discriminate between these apparently very similar ions *in vivo*. There is a cluster of nine silver resistance genes (*sil*), *silABCEFGPRS*, found in many Gram-negative bacteria (1, 10, 11, 15, 16). Gene products include SilABC, an RND+ efflux pump and membrane complex that spans the inner and outer membranes; SilP is an inner membrane Type P_1B_-ATPase; SilR and SilS form a two component signaling system that controls inducible silver resistance. The proteins SilE and SilF are periplasmic chaperones, while the role of SilG is so far unknown. Previously, we have characterized Ag(I) binding to SilE, showing that it is a disordered protein that folds upon binding six Ag(I) but can bind up to eight ions (17). We now turn our attention to the chaperone SilF.

It is known from work on the copper resistance mechanism that the SilF homolog, the periplasmic chaperone CusF, binds Cu(I) and Ag(I), but not Cu(II) (18). It is responsible for shuttling Cu(I) to the CusABC efflux complex for export out of the cell. Recent evidence has also emerged that CusF is prevented from oxidation of its ion-binding methionine residues by MsrPQ, enabling it to remain active in the more oxidizing environment of the periplasm (19). It is likely that SilF performs a similar role as a metallochaperone (11).

In this paper, we characterize structurally, biophysically, and by simulation the relative Ag(I)- and Cu(I)-binding properties of SilF from *E. coli* and show how measurement of the metal ion specificity illuminates the biological role of the *sil* system.

## Results

### The structures of apo and holo SilF

**Figure 1:**
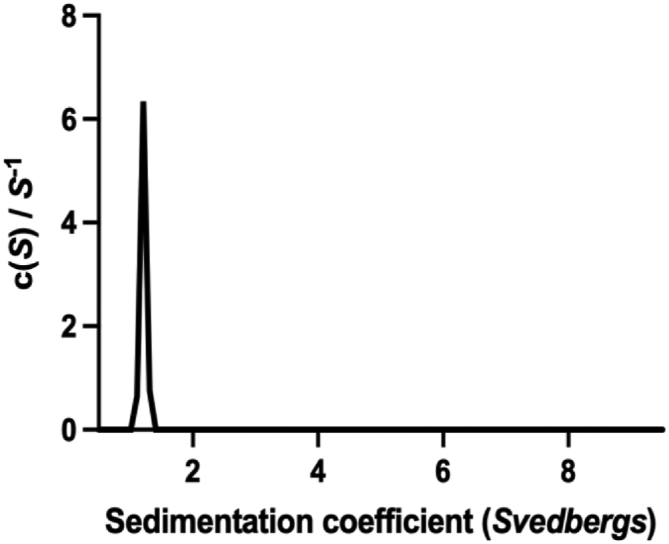
**Sedimentation coefficient distribution derived from sedimentation velocity analytical ultracentrifugation of apo-SilF**_**38-120**_**.** The protein sediments as a monodisperse species with no higher order aggregates observed. A similar result is found for both the Cu(I)- and Ag(I)-bound forms (data not shown).

**Figure 2:**
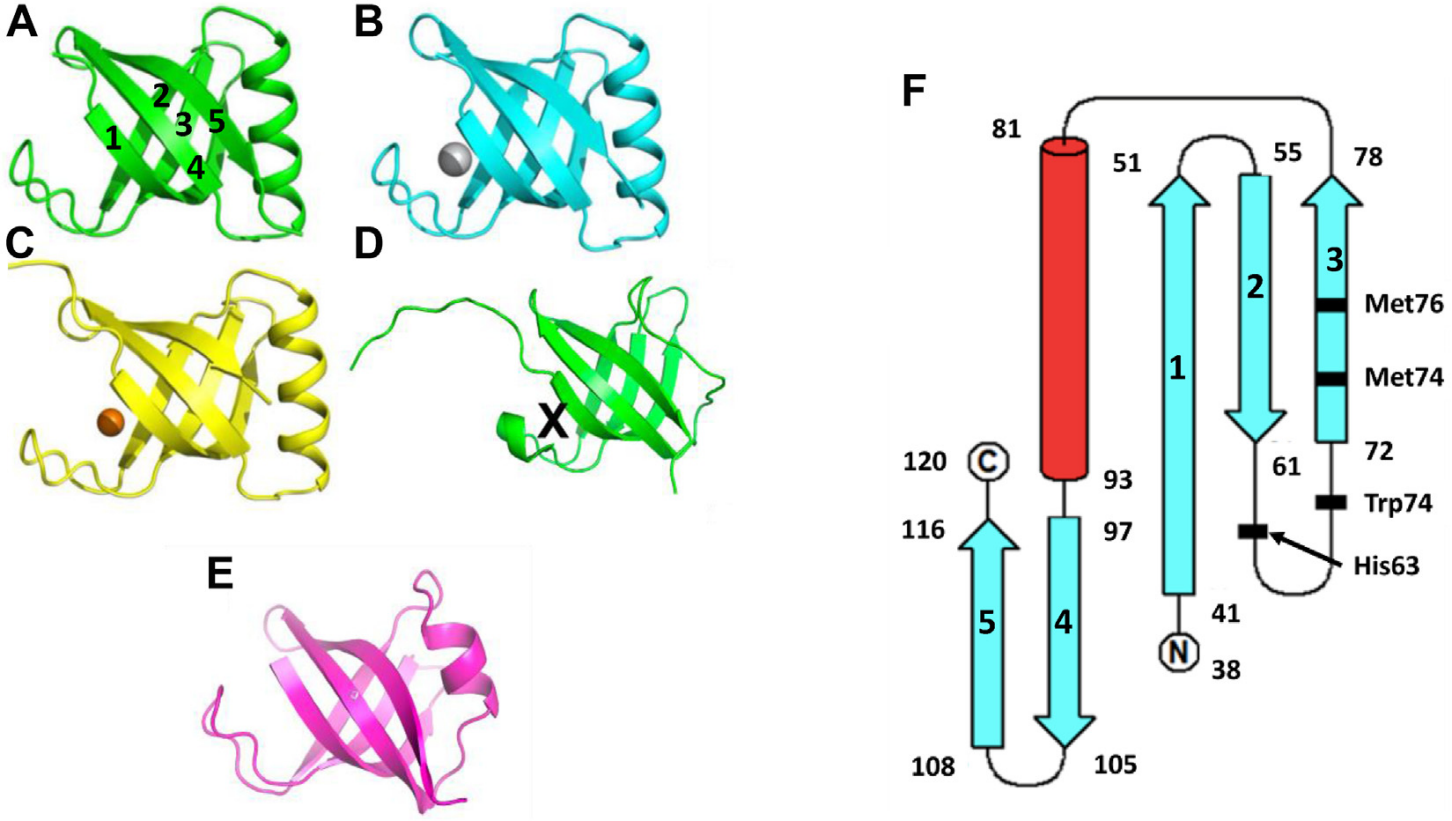
**Ribbon diagram representation of protein structures.***A,* apo- SilF_38-120_ (PDB: 8BBZ), (*B*) Ag(I)- SilF_38-120_ (PDB: 8BHU), (*C*) Cu(I)- SilF_38-120_ (PDB: 8BI1), and (*D*) CusF (PDB: 2VB3). The ion-binding site in CusF is shown by X. *E*, AlphaFold2 prediction of SilF. The prediction for the structure shown is at the level of pLDDT > 90%; the predicted disordered signal peptide has been omitted. *F*, topology of SilF showing the residues involved in ion binding marked in *bold*. In both *A* and *F*, the numbering of the strands is shown for clarity.

**Figure 3:**
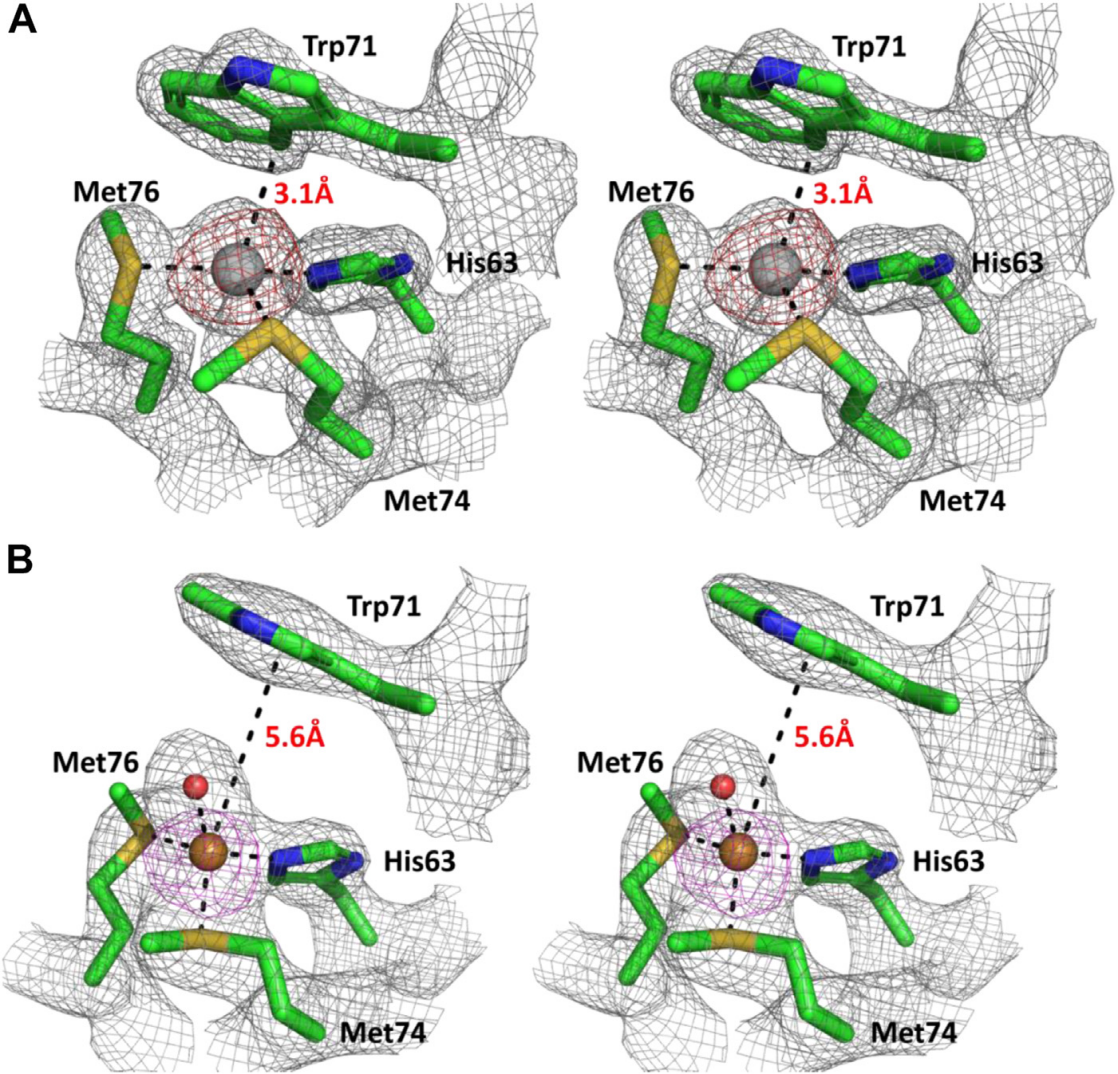
**Wall-eye stereo view of cation-binding site in SilF**_**38-120**_**.** Residues involved in (*A*) Ag(I) and (*B*) Cu(I) binding in SilF_38-120_. Electron density at 1.5 σ from refined 2F_0_-F_c_ maps is overlaid for information; *red* is the anomalous density. The Cu(I) ion is smaller (0.60–0.74 Å) than the Ag(I) (1.0–1.14 Å), and as such, an extra water molecule is coordinated in the binding site.

The first 37 residues of SilF contain a periplasmic export sequence as well as a short predicted disordered region. Therefore, we cloned and expressed SilF from residues 38 to 120. The molecular oligomerization state of SilF_38-120_ was first investigated by size-exclusion chromatography coupled to multi-angle light scattering (SEC-MALS) (Fig. S1) and sedimentation velocity analytical ultracentrifugation (Fig. 1). Single species were observed by both techniques. The molecular weight of SilF_38-120_ was determined to be 8.74 kDa (±7.9%) by SEC-MALS (Table S1) and 9.0 (±0.2) kDa by sedimentation velocity analytical ultracentrifugation. Both of these values are consistent with the calculated molecular weight of 9.1 kDa, indicating that SilF_38-120_ is monomeric in solution; neither method detected any higher order aggregates. Next, we determined the structure of SilF_38-120_ using X-ray protein crystallography (Fig. 2*A*). Apo-SilF38-120 packed in a hexagonal unit cell with one protein chain per asymmetric unit. The protein has a β-barrel topology composed of five β-stands arranged in the β1-β2-β3-β5-β4-β1, similar to that observed for Cu(I) chaperone CusF (20) (Fig. 2*D*). Unlike CusF, SilF_38-120_ has an additional 15 amino acid α-helix positioned between strands β3-β5. In CusF, this is an extended loop with no discernable helical or sheet secondary structural elements (See Fig. 2*D*). Interestingly, the prediction from AlphaFold2 (Fig. 2*E*), which will have been trained using CusF, but not our structures, does show a shorter helix around 50% of the size of the one observed in SilF_38-120_ in this position; the rest of the residues are predicted to be disordered. Cocrystallization of SilF_38-120_ with either Ag(I) or Cu(I) under anaerobic conditions yields an orthorhombic unit cell with three protein chains per asymmetric unit (Table S2) and each chain with one metal ion bound (Fig. 2, *B* and *C*) close to one end of the β-barrel (Figs. 2 and 3). We attempted cocrystallization with Cu(II) but were unsuccessful. Calculation of the sedimentation coefficient using the coordinates of each of the monomers (21, 22, 23) taken from the respective crystal structures yielded a value of 1.25 *S*, consistent with a monomer of SilF_38-120_ measured by sedimentation velocity. In both structures, the metal ion is tetrahedrally coordinated (Fig. 3), and metal ion binding appears to have a limited effect on the overall conformation of the protein. RMSDs derived from the C_α_ atoms are 0.99 Å for Ag(I)/Apo and 1.09 Å for Cu(I)/Apo. Larger deviations are observed primarily in the metal-binding site, the top of the α-helix, and the final loop that leads into the C terminus. Recalculating RMSDs with these two regions missing reduces the value to 0.68 Å for Ag(I)/Apo and 0.98 Å for Cu(I)/Apo, showing that changes in flexibility is confined to the binding site and these regions. There are distinct conformational differences of residues at the metal ion–binding site and the loop connecting β4-β5. In both the Ag(I)- and Cu(I)-bound structures, the metal ion is coordinated with distorted tetrahedral geometry through the donor groups NE2 of His63 and the two thiol groups of Met74 and Met76. Figure 3 (both panels and Table S3) show the bonds and their lengths between the residues and metal ions for both SilF_38-120_. A comparison with CusF (18) (PDB: 2VB3, Table S3) shows that the metal ion coordination distances are identical. In SilF_38-120_, Trp71 acts as a cap over the metal coordination site, which likely further stabilizes binding of the Ag(I) *via* cation–π interactions between the aromatic indole ring system and the positively charged metal ion.

The Cu(I) coordination geometry within the binding site is noticeably different than that of Ag(I) where the fourth coordination position of the Cu(I) ion is occupied by a water molecule. The Trp71 is now blocked from directly interacting with the metal. Furthermore, the side-chain of Met74 adopts a different rotameric conformer compared to the apo and Ag(I) structures. Such flexibility allows the protein to provide ligands at shorter coordination distances so as to accommodate the smaller ion. By contrast, the conformations of Met76 and His63 remain unchanged between structures. This difference in the observed water coordination for SilF_38-120_-Cu(I) binding is again notably different to CusF-Cu(I) binding which demonstrates close coordination of the equivalent Trp. It is argued that this interaction and connected water exclusion from the binding side is important to prevent Cu(I) oxidation when bound to CusF((20, 24, 25, 26)).

Structural homology searches (27) revealed that, in addition to CusF, the subunit S1 of pertussis toxin (PDB 1PRT), a domain from pro-protein glutaminase (PDB 3A54), and subunit B of subtilase cytotoxin (PDB 3DWA) were most similar in structure to SilF_38-120_. These structures are either domains or small proteins classified as oligonucleotide/oligosaccharide binding (OB) fold proteins that are typically found in oligonucleotide/OB domains. They are comprised of five or more β-strands interlinked with either an α-helix, extended loop, or a three-helix bundle between strands β3-β4((28, 29, 30)). The binding regions of OB-fold proteins vary with no singular defined binding region with different proteins using different loops at either end of the barrel to bind their target ligand (31, 32). The molybdenum sensor ModE that regulates transcription of several genes involved in cellular molybdenum homeostasis is currently the only other example of a metal binding OB-fold protein (33), although here metal binding is a prerequisite for binding ssDNA((34)). SilF and CusF are therefore the only examples of OB-fold proteins with the sole function of metal ion binding.

### Conformational flexibility

**Figure 4:**
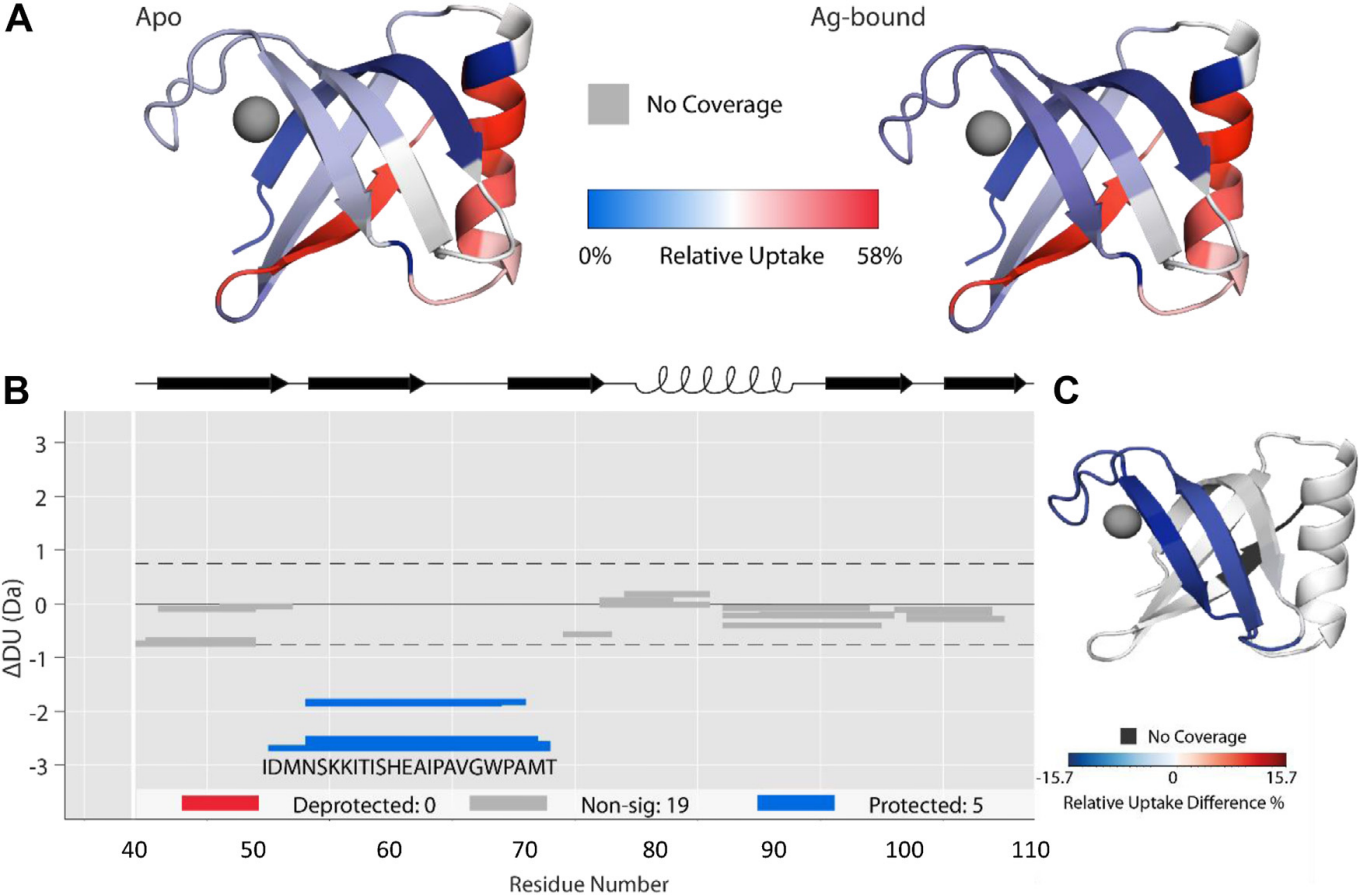
**Changes in peptide deuterium uptake over time measured by HDX-MS.***A*, the individual deuterium uptake of both the apo and Ag-bound states of SilF_38-120_ overlaid onto the crystal structure. *B*, wood differential plot showing statistically relevant changes (hybrid significance testing *p* < 0.001; *Deuteros* (59)) in deuterium uptake after 30 min of incubation in D_2_O. *C*, wood differential overlaid onto SilF structure. *Blue* indicates a decrease in deuterium uptake upon Ag binding; *red* for an increase. Changes are confined to the beta strand 2 and 3 and the binding site loop. HDX-MS, hydrogen-deuterium mass spectrometry.

Hydrogen-deuterium mass spectrometry (HDX-MS; Fig. 4) was used to probe changes in conformational flexibility over time (30s, 5 min, & 30 min) and to provide localized in-solution evidence of metal binding to SilF_38-120_. A coverage map was generated, covering 88.9% of the protein amino acid sequence (Fig. S2). The deuterium uptake data is able to illustrate localized differences in conformational flexibility. While the majority of the beta-barrel resists deuterium uptake due to the high number of hydrogen bonds involved in the secondary structure, beta-strand 5 (C-terminal) exchanges readily, indicating increased solvent exposure compared to the remaining beta strands. Similarly, the alpha helix exchanges readily and suggests flexibility within the helix structure.

Upon incubation with Ag(I), there are significant changes in deuterium uptake on peptides that span the amino acid sequence IDMNSKKITISHEAIPAVGWPAMT (residues 52–75), which encompasses the known metal-binding site (binding residues shown in bold). The observed reduction in relative deuterium uptake (15.7%) indicates that upon binding, Ag(I) interacts with amino acids within this region of the protein, including His63, Trp71, and Met71 in agreement with the metal coordination site determined by the crystal structure, and blocks the amino acids from exchanging with solvent deuterium. Due to experimental set up, we were unable to carry out Cu(I) binding in suitable anaerobic conditions.

**Figure 5:**
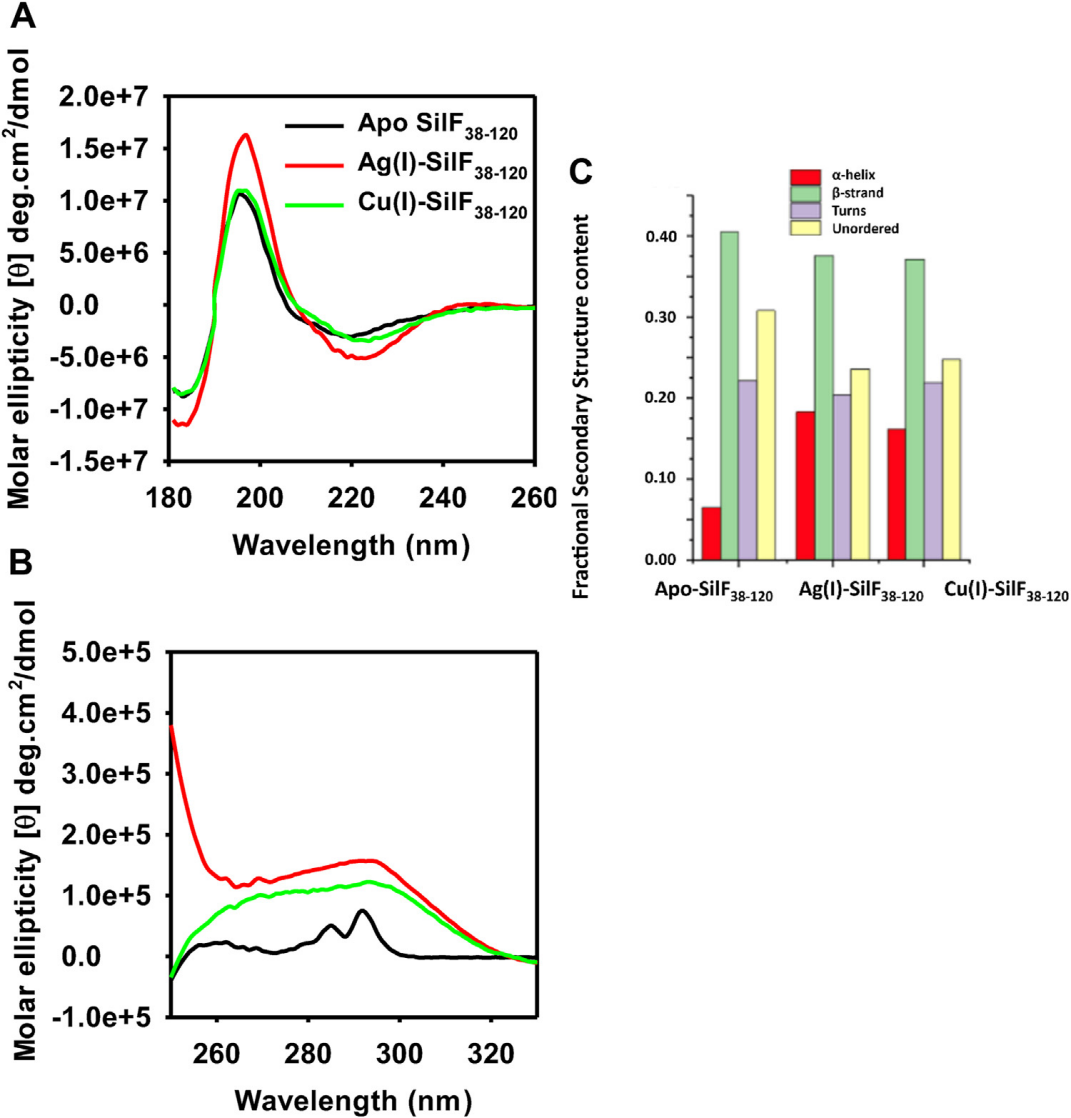
**Circular dichroism spectra of SilF**_**38-120**_**in the apo, Cu(I)-, and Ag(I)-bound forms.***A*, far UV CD showing changes to secondary structure. *B*, near UV CD showing changes to aromatics residues upon ion binding. *C*, results of secondary structure deconvolution of synchrotron radiation circular dichroism (CD) of SilF_38-120_ in the apo, Cu(I)-, and Ag(I)-bound forms. Experimental conditions are in the supplementary materials.

To assess any changes in secondary structure that could occur in ion binding, we employed CD spectroscopy. There were distinct differences in CD spectra in both the far and near UV regions (Fig. 5, *A* and *B*). Decomposition of the far UV region into secondary structure elements (Fig. 5*C*) showed that upon addition of both metal ions, there is an increase in α-helical content from 6% to 16% at the expense of disordered protein. Although changes in RMSD between the structures are observed (see above), there is little apparent change in helicity, although this may be due to crystal packing. Near-UV CD spectra, measuring the impact on aromatic residues, showed that upon addition of both Ag(I) and Cu(I), there were large spectral differences consistent with changes to Trp71 in the metal-binding site (Fig. 5*B*). It is noticeable that the Ag(I)/SilF_38-120_ complex has a near-UV CD spectra that is distinct from the Cu(I)–SilF_38-120_ complex. This we attribute to the differences in binding mode of the Trp71 residue seen between the two crystal structures.

### Metal ion binding and specificity of SilF

In order to assess the affinity and thermodynamics of metal ion binding, we performed isothermal titration calorimetry (ITC) measurements. Studying binding events of Cu(I) in aqueous media is challenging for two reasons: (i) under aerobic conditions, Cu(I) readily oxidizes by reacting with O_2_ from air, to give Cu(II); (ii) under anaerobic conditions, Cu(I) undergoes a disproportionation reaction, resulting in the formation of Cu(0) and Cu(II) (Equation 1) (35).

(eq1) $$2Cu_{\left(\text{aq}\right)}^{+}⇌\;Cu_{\left(\text{aq}\right)}^{2+}+Cu_{\left(s\right)}^{0}$$

**Figure 6:**
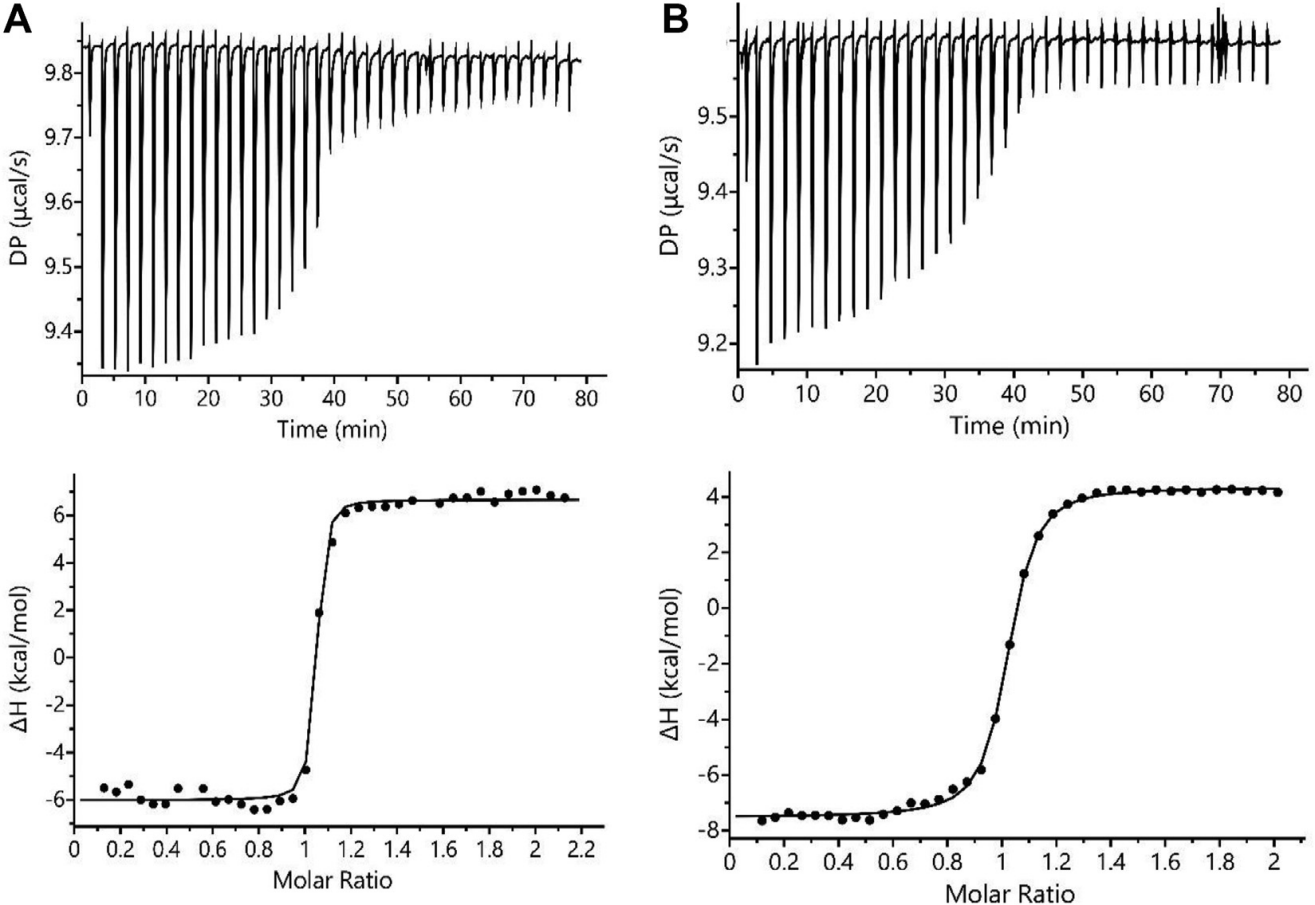
**Isothermal titration calorimetry data.***A,* Ag(I) *versus* SilF_38-120_ and (*B*) Cu(I) *versus* SilF_38-120_. The *upper panels* are the raw thermograms, and the *lower panels* show data fitted to a 1:1 binding model to the integrated heat data per injection. ITC, isothermal titration calorimetry.

An excess of NaCl in solution can prevent the disproportionation of Cu(I) from occurring under anaerobic conditions, thus we performed all Cu(I) titrations at 1 M NaCl (36). This equates with what was previously used for CusF/Cu(I) titration (37). Solubility of AgCl is poor, resulting in precipitation even in the presence of moderate concentrations of NaCl, preventing the use of identical salt concentrations for both experiments. Interactions of Cu(I)/Ag(I) with buffer molecules are considered to be weak. However, due to the relatively high concentration of buffer compared to the binding metal, a non-negligible effect of the buffer molecules on binding was observed, as has been reported previously for such ion titrations (38, 39). To avoid these issues, the titrations were performed in the absence of buffer. We found that the binding of either metal to SilF_38-120_ has a 1:1 stoichiometry, confirming the observations from the crystal structures of a single binding site. Binding was exothermic (Fig. 6) with a small entropic penalty (Table 1), which accords with the changes in flexibility and helicity seen in CD measurements. Ag(I) has a dissociation constant (*K*_d_) of 7.6 nM, whereas Cu(I) binds with a *K*_d_ of 30 nM: both determined free energies values were within experimental error of each other meaning that the affinity of SilF_38-120_ for either metal ion are very similar in size. Previous investigations using ITC of CusF under anaerobic conditions observed that Cu(I) was bound considerably more weakly than Ag(I) (37). These experiments also yielded low estimates for stoichiometry of binding (0.52 for a protein known to bind with 1:1 stoichiometry).

**Table 1:** Thermodynamic parameters derived from isothermal titration calorimetry experiments for SilF_38-120_ binding with Ag(I) and Cu(I)

Expt	*K*_d_ (nM)	Stoichiometry (*n*)	Δ*H*° (kJ/mol)	Δ*S*° (J/mol/K)	Δ*G*°(kJ/mol)
SilF_38-120_/Ag(I)	7.6 (± 1.3)	0.99 (± 0.03)	−55.1 (± 1.59)	−29.0 (± 1.7)	−46.4 (± 0.5)
SilF_38-120_/Cu(I)	30.0 (± 6.5)	0.96 (± 0.22)	−54.8 (± 1.1)	−34.0 (± 3.9)	−44.6 (± 3.7)
CusF/Ag(I)	38.5 (± 6.0)	0.52 (± 0.08)			
CusF/Cu(I)	495 (± 260)	0.82 (± 0.09)			

Data for CusF affinity and stoichiometry is taken from (37): no other thermodynamic data was given in this reference.

### Molecular dynamics and QM/MM analysis

**Figure 7:**
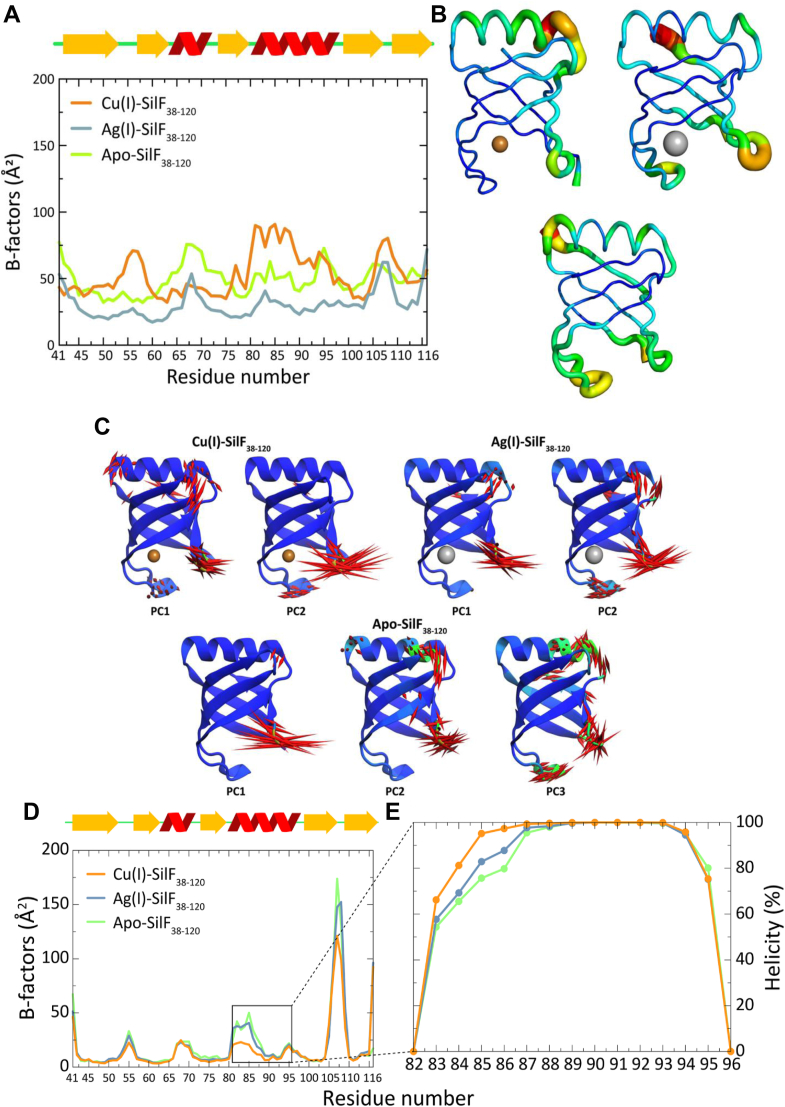
**Comparison of flexibility data derived from experiment and simulation.***A*, experimental B-factors for Cu(I)-SilF_38-120_, Ag(I)-SilF_38-120_, and apo-SilF_38-120_. *B*, experimentally derived structures of Cu(I)-SilF_38-120_, Ag(I)-SilF_38-120_, and apo-SilF_38-120_ showing the magnitude of the B-factors as a tube plot. *C*, normal mode displacements (larger than 2 Å) of first most prominent principal components. Displacements indicated in *red* by magnitude; those with high displacement along the helical axis in PC2 and PC3 of apo-SilF_38-120_ are indicated in *green*. The data was collected from 1.6 μs classical MD simulations of Cu(I)-SilF_38-120_, Ag(I)-SilF_38-120_, and apo form. *D*, residual root-mean-square fluctuation (RMSF) of SilF_38-120_ backbone atoms (N, Cα, C, O) in Cu(I)-SilF_38-120_ and apo-SilF_38-120_ form calculated from the reference X-Ray structure of apo protein. *E*, secondary structure features of α-helix calculated for Cu(I)-SilF_38-120_ and apo form calculated using the database of secondary structure assignments (DSSP) algorithm, which assigns average secondary structure propensities over MD frames for each residue based on backbone amide (N-H) and carbonyl (C=O) atom positions. The protein was truncated in the simulations at the N and C termini to minimize fluctuations. All simulation data, a total of 1.6 μs per system, was used to generate the RMSF for each system from which *B* factors were calculated as per equation in Experimental procedures.

To further probe the changes in conformation upon metal ion binding and computationally investigate differences in binding affinity of the different metal ions, classical molecular simulations and Quantum Mechanics/Molecular Mechanics (QM/MM) hybrid methods were employed. We used our determined crystal structures for Apo-SilF_38-120_, Ag(I)-SilF_38-120_, and Cu(I)-SilF_38-120_. We were unable to investigate a Cu(II)-SilF_38-120_ binding due to the previously described failure to produce crystals for this complex. Initial QM/MM optimizations matched well the structural differences observed for the different metal ion binding, including the addition of a water molecule in the coordination of Cu(I). Subsequent long (1.6 μs) timescale molecular dynamics (MD) simulations confirmed conformational changes upon binding as seen in the HDX-MS results as demonstrated by principal component analysis (Fig. 7*C*). There were changes in flexibility seen in the α-helix correlating well with changes in secondary structure contents indicated by CD (Fig. 5), again indicating that the lack of changes in helicity observed in the crystal structure may arise from crystal packing. Visualization of the dominant principal components and the helicity analysis from the simulations shown in Figure 7, *D* and *E* show changes in conformation, as previously observed with HDX-MS and CD. Analysis of the metal ion–binding site from those simulations also confirmed the longer stability of the binding geometry in the ion-bound complexes as well as differences between Ag(I) and Cu(I) binding.

Experimentally derived B-factors from crystallographic data are shown in Figure 7, *A* and *B*. It can be seen from comparison of Figure 7, *A* and *D* that the variation in B-factors reflects the general trend in flexibility in both the experimental and simulation data, with increased flexibility seen between residues 50 to 60 and 65 to 75, both spanning the ion-binding site, and correlate well with the changes seen in HDX-MS (Fig. 4). Additional changes in flexibility are seen in the helix region as noted above and also residues 105 to 110 which reflect changes in the C terminus which is situated adjacent to the helix. The simulation data shows a greater change in this latter region than the experimental data; however, this maybe due to crystal packing factors.

**Figure 8:**
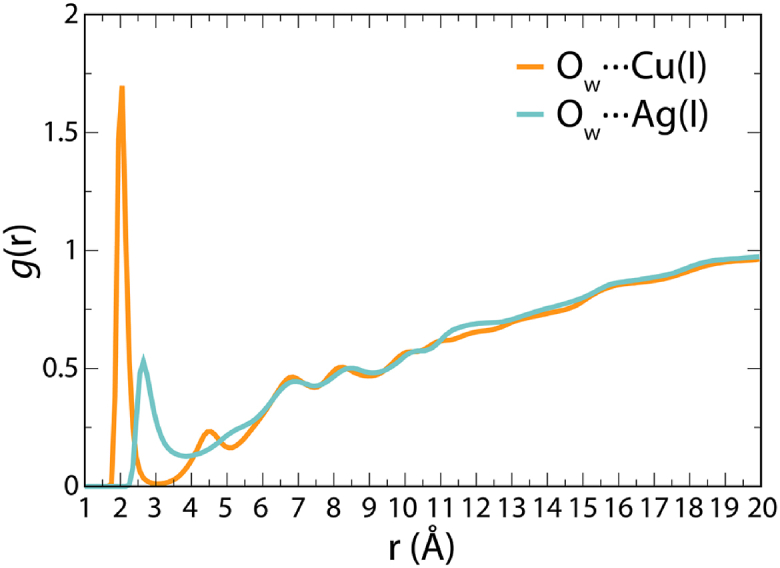
**Radial distribution function of water oxygen atoms around metal ions.** The data was collected from 1.6 μs classical MD simulations.

As shown in Figure 8 by the radial distribution, Cu(I) retains tightly bound water molecules in its tetrahedral coordination sphere throughout the whole simulation. In comparison, while interactions of Ag(I) with water molecules could also be observed, they appeared at a longer distance which allows Trp71 to bind closer to the metal.

**Figure 9:**
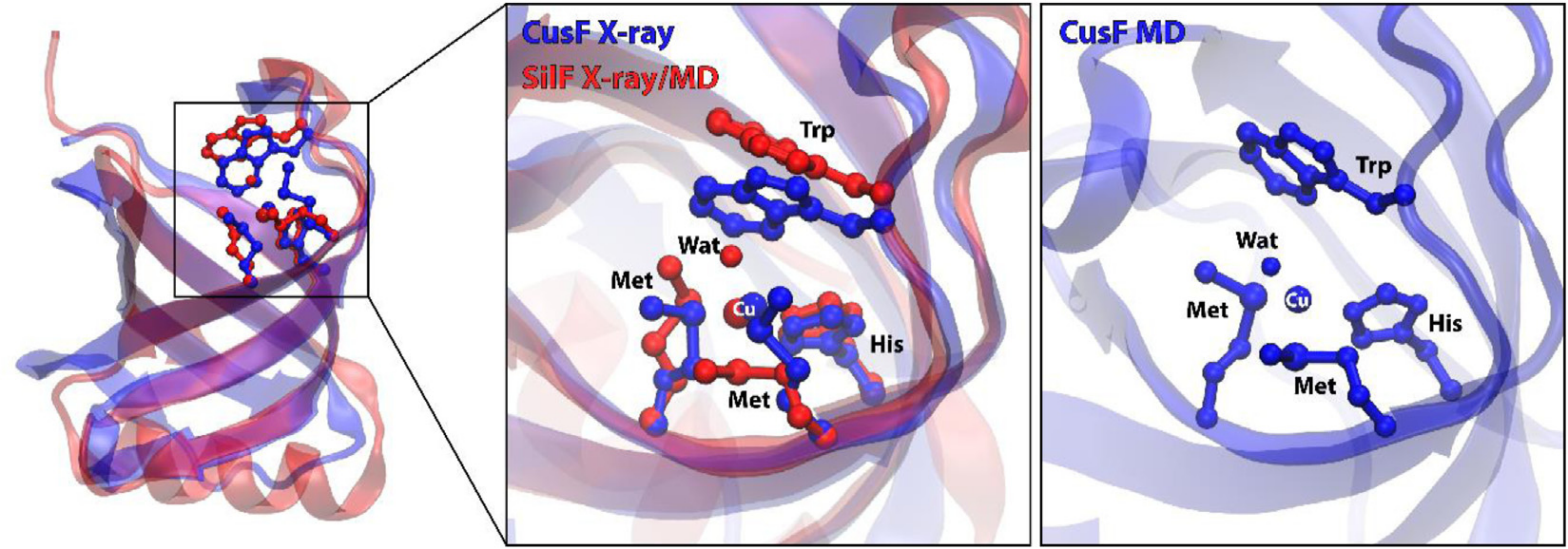
**Comparison of different binding modes obtained for Cu(I) in CusF and SilF**_**38-120**_**F.** In X-ray structure of CusF with Cu(I) (*blue*), the sidechain of Trp is found closer to the copper while the structure of SilF_38-120_ demonstrates the coordination of a bound copper by one water molecule (*red*). A similar structure is obtained in MD simulations for both CusF and SilF_38-120_ whereby water molecules have strong preference towards coordinating copper in its bound state.

In contrast, the crystal structure of Ag(I)-CusF does not display a bound water molecule (20). However, when we carried out a MD simulation of CusF-Cu(I), this showed a water molecule present, coordinating between Trp71 and Cu(I) (see Fig. 9) as seen in our Cu(I)-SilF_38-120_ crystal structure (see Fig. 3). Previous simulation studies were able to replicate the absence of this water molecule in CusF by reparametrizing the Lennard-Jones potential to increase the strength of the Cu(I)–π interaction. When this study used a traditional potential, 60% occupancy of water akin to what is seen in the Cu(I)-SilF_38-120_ structure was observed (40). Coordination between Trp71 and Cu(I), as shown in Figure 9 and as demonstrated by the sharp peak in the radial distribution function of water around the metal ions, is presented in Figure 8.

The residency times for water in the first hydration shell around the binding site of Cu(I)-SilF_38-120_ is on average 101 ps with an SD of 117 ps. However, for Ag(I)-SilF_38-120_, this value was considerably shorter with an average of 5 ps and an SD of 8 ps. (Solvation shells are defined as the area under the first solvation peak as seen in the radial distribution function in Fig. 8). Therefore, judging from the combination of those different sets of simulations and the crystallographic evidence of the two binding sites, it appears that the binding site of SilF is adaptable with at least two different possible modes of binding including the observed water coordination, differently to the CusF-binding site where water exclusion dominates.

To further probe the changes in SilF protein conformation upon metal binding, multiscale modeling was applied based on structures from the extensive classical MD simulations. For this analysis, over 40 ps of QM/MM simulations starting from equilibrated MD simulations were performed.

**Figure 10:**
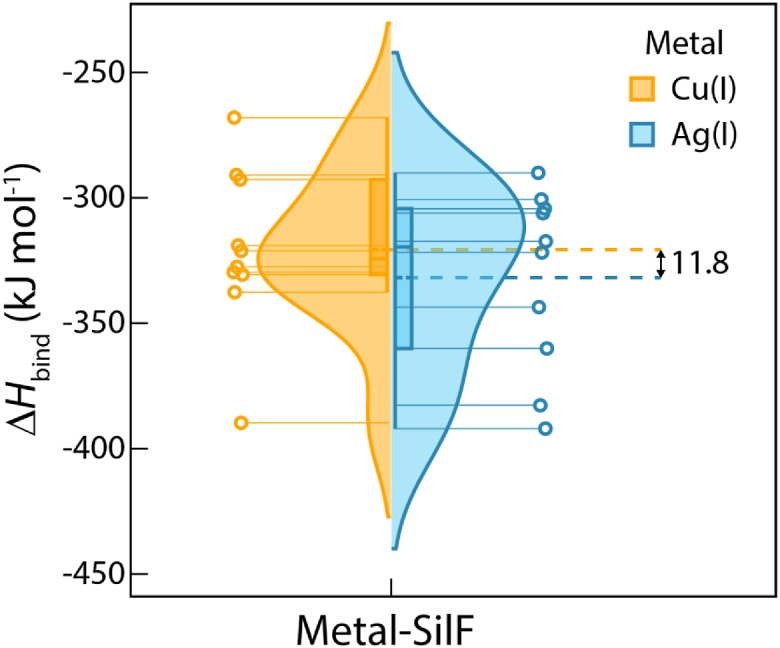
**Violin plot representation with box and discrete data points of binding enthalpies for Cu(I)-SilF**_**38-120**_**and Ag(I)-SilF**_**38-120**_**obtained from ONIOM calculations using ten different structures from QM/MM MD simulations.** Although a slightly higher enthalpy is calculated for Ag(I) binding, this is not significant within the wider spread of calculated enthalpies where the standard deviations for Cu(I) and Ag(I) binding were 32.86 kJ/mol and 35.88 kJ/mol, respectively. A violin plot contains box plot (median, interquartile range and *upper*/*lower* half shown as orange and *blue lines* inside the box for Cu(I) and Ag(I), respectively) with the addition of a rotated kernel density plot for each system. The quartiles Q1 and Q3 are computed using the linear interpolation method. A difference between average values of D*H* for Cu(I) and Ag(I) is depicted with *dashed* lines. QM/MM, Quantum Mechanics / Molecular Mechanics.

Those simulations demonstrated stable ion-binding coordination, and ten snapshots have been picked to calculate the binding enthalpies of the different ions relative to their solvation enthalpies in water (see Supporting Information for methodology). The binding enthalpies calculated with static QM/MM methodology demonstrated large variations, and although a slightly higher average binding enthalpy for Ag(I) by 11.8 kJ/mol is calculated, (Fig. 10), the SD of each of the enthalpy calculations is around three times this value, making this enthalpy difference between the two binding events statistically insignificant. This is also in line with the experimental ITC measurements (see Table 1) where changes in enthalpy and free energy changes cannot be distinguished statistically for both ions.

## Discussion

Biophysical and biochemical analysis of proteins comprising the *sil* gene cluster has been limited to date, with sequence homology used to infer function and the proposed resistance mechanism, after comparison with the more extensively studied *cus and cue* systems (1, 10). Such analysis is consistent with the model that the SilF is a periplasmic metal-binding chaperone capable of binding both Ag(I) and Cu(I) ions and shuttling them to the SilCBA complex to aid metal ion detoxification (41, 42).

Our analysis supports that the observed lack of any appreciable metal ion–binding preference of SilF for Ag(I) compared to Cu(I) which was evident from the ITC measurements. The dissociation constant of 7.5 nM for Ag(I) is a little tighter than the affinity measured for CusF (38 nM) (37), whereas the relative Cu(I) affinities of SilF (30.0 nM) and CusF (450 nM) differ by more than an order of magnitude (15, 37). The significant difference is in the binding stoichiometries, 0.5 for CusF(see (37)) and 1.0 for SilF (this study), despite the crystal structures of both showing a single ion binding to the monomer.

The X-ray crystal structures show that the metal ion–binding site is formed from strictly conserved residues His63, Trp71, Met74, and Met76 located at one end of the β-barrel. Upon Ag(I) binding, histidine and methionine residues occupy three of the coordination sites of the bound metal. The coordination sphere is completed by the indole ring of Trp71, which forms a π-cation interaction to complete the tetrahedral coordination sphere. On binding of Cu(I) His63, Met74 and Met76 again adopt a distorted trigonal coordination geometry, but the fourth coordination site is occupied by a water molecule, preventing the formation of the π–cation interaction. MD shows that this persistence of the water molecule suggests the π–cation interaction with the Cu(I) ion is insufficiently stable to displace the solvating water molecule. The slightly more favorable entropy change observed by ITC for Cu(I) binding does point towards the participation of water in this interaction. However, simple thermodynamics arguments are not enough to explain the presence of the water molecule since it does not persist in the CusF-Cu(I) structure, which has a highly similar active site architecture.

The inability of SilF to form a π–cation interaction with Cu(I) will decrease the binding enthalpy for copper, a further reduction in binding enthalpy will occur from a preference of sulfurous ligands to bind Ag(I) relative to Cu(I) (43). The high-resolution structure of CusF with Ag(I) bound (18, 20) shows the coordinating methionine residues can adopt multiple conformations while still interacting with the metal ion. Such freedom of movement within the relatively flexible coordination sphere of Ag(I) reduces the entropic penalty of metal binding. The smaller ionic radius of Cu(I) (0.60–0.74 Å) relative to Ag(I) (1.0–1.14 Å) constrains the geometry of the coordinating methionines as they tuck into the binding site and interact with the metal. Stabilization of Cu(I) *via* a π–cation interaction has previously been demonstrated for Cu(I) binding to CusF (18) and is likely the cause of the difference in Cu(I)-binding affinity between CusF and SilF. The adaptability of the binding site leading to this lack of ion specificity is also supported by results from QM/MM binding enthalpy calculations.

Displacement of the tryptophan loop is not the only conformational change observed within SilF. HDX-MS shows changes globally across the protein, and CD spectroscopy show an increase in alpha helical content of SilF upon cation binding. Although we do not see appreciable changes in helix formation from the crystal structures, most likely due to crystal packing forces, both of the solution experimental observations are well supported by the simulations where changes in helicity are clearly over long simulation times between the apo and holo SilF. Together, these show that the capping helix is unstable in the apo-protein and indeed could be unfolded to a degree in solution, as observed for CusF, with metal binding stabilizing the helix by an as-yet undefined allosteric mechanism. Since residues involved in the CusB–CusF interaction (44) have been identified at this end of the barrel, it is possible that metal binding stabilizes an SilB-binding site to aid docking of the metallochaperone to the SilABC efflux complex.

## Conclusion

Ag(I) is a potent antimicrobial and a major part of its bactericidal action arises from its ability to mimic Cu(I) as a preferred binding partner to copper-binding proteins. Our studies show that key changes in the flexibility of SilF shown by solution-based techniques, but not by crystal structure analysis, indicates that our crystallization conditions impact on the helical content of the protein. The differing role of water in the two binding mechanisms allows an adaptability of the binding site to accommodate the two different cations with similar affinity.

The efficiency with which Ag(I) can be removed from bacteria is essential for biological function in these organisms as this ion serves no biological purpose. Evidence is now emerging that carriage of *sil* genes, either on the chromosome or *via* a plasmid, is not a prerequisite for Ag(I) resistance. It is a mutation in the *silS* gene (15, 45) leading to an upregulation of the *sil* genes that then leads to the observed enhanced resistance. Hence, with our observations of the similar affinity for both cations, it is an increase in expression of the Sil proteins that leads to the observed Ag(I) resistance, rather than simply the expression of proteins that have a higher affinity for Ag(I). Our findings therefore logically lead to the hypothesis that there is not anything particularly novel about the Sil proteins compared with the Cus proteins: resistance simply arises from there being more Sil proteins available to bind Ag(I) in resistant strains compared to nonresistant strains: the *sil* genes are frequently found with the *pco* genes on a Tn7-like mobile genetic element.

Given the results reported here, this leads to an intriguing and more general hypothesis that there is no specific Ag(I) resistance mechanism; there is a Cu(I) resistance mechanism and metallochaperone function that can accommodate both Ag(I). Our increased release of Ag(I) into the environment (4, 5, 6, 7) and the apparent observed rise in Ag(I) resistance (41, 46, 47) has now to be looked at in a different light. This now appears to arise from the lack of discrimination between Cu(I) and Ag(I) by the existing Cu(I)-resistance mechanisms, rather than the evolution of a new mechanism. The consequences of this for how we define a particular metal resistance mechanism and understand their evolution in the environment are therefore profound (48).

During this manuscripts submission and revision in the light referees comments the NMR-derived structures of apo-SilF and SilF-Ag(I) were published (49). This showed a shorter helix more akin to the structure of CusF; however, due to the coordinates being on hold, we are unable to do any direct comparison and only note this now as an intriguing observation worthy of further investigation. NMR titrations published in that manuscript showed a micromolar dissociation constant, some 2 to 3 orders of magnitude weaker than that found by us in this study. The reason for this appears to be that the affinity in this study was measured by NMR at concentrations several orders of magnitude above the dissociation constant and therefore close to the tight binding limit. A full discussion of this phenomena can be found in (50).

## Experimental procedures

### Cloning, expression, and purification

The SilF gene from *E.coli* (Uniprot: A0A3T0VBZ2) was synthesized by Twist Bioscience as a gene fragment. Analysis of the construct indicated the first 38 amino acids formed a disordered region; therefore, a truncated version (SilF_38-120_) from V38 was PCR amplified from the full-length sequence. The amplified gene was cloned into a pOPINF vector (PPUK, Rosalind Franklin Institute,) using PPUK’s in-fusion method; the vector contains an N-terminal His6-tag with a HRV3C cleavage site (51).

pOPINF-SilF_38-120_ was transformed into BL21 (DE3) *E. coli* cells for expression. Overnight precultures were prepared using a single colony grown in 20 ml TB media supplemented with 20 μl of ampicillin (50 mg/ml), grown at 25 °C. Precultures were used to inoculate 1 L of TB media, supplemented by 1 ml of ampicillin (50 mg/ml), using 10 ml of preculture per liter. Cultures were grown at 37 °C with shaking until an OD (600 nm) of 1.2 was achieved, whereupon 1 ml of 1M IPTG was used to induce, the cultures were left to grow at 22 °C for 16 h.

Cells were harvested at 5000*g* for 10 min with the subsequent pellets resuspended in lysis buffer (50 mM Hepes, 500 mM NaCl, 20 mM imidazole, pH 7.8), using 50 ml per 10 g pellet. Lysis buffer was supplemented with lysozyme (0.1 mg/ml), DNase I (0.1 mg/ml), and 1 Roche cOmplete protease-inhibitor cocktail tablet (EDTA-free). Cells were lysed using a cell disruptor with two passes at 28kpsi; the resulting lysate was centrifuged at 40,000*g* for 30 min. The supernatant was run down a 5 ml Ni^2+^ His-Trap column pre-equilibrated with lysis buffer using a peristatic pump; after loading, the column was washed with 20 CV wash buffer (50 mM Hepes, pH 7.8, 500 mM NaCl, 50 mM imidazole). A gradient elution was used to elute SilF using buffer A (50 mM Hepes, 500 mM NaCl pH 7.8) and buffer B (50 mM Hepes, 500 mM NaCl, 750 mM imidazole pH 7.8); the length of elution was 20 CV with an end concentration of 100% buffer B, 3 ml fractions were collected.

Fractions containing SilF_38-120_ were pooled together for dialysis; 3C protease and β-mercaptoethanol (5 mM end conc) were added to the sample. Samples were dialyzed (Spectra/Por3 RC Tubing, MWCO 3.5 kDa) overnight against SEC buffer (25 mM Hepes, pH 7.8, 150 mM NaCl). Dialyzed material was run down a reverse IMAC Ni^2+^-HisTrap, with samples coming off in the flow through and partially in the wash. SilF was concentrated down to a volume of 1.5 ml and loaded onto a HiLoad 16/600 Superdex 75 PG column, pre-equilibrated with SEC buffer, with 3 ml fractions collected. SilF_38-120_ presence was confirmed by SDS-PAGE analysis (with approximately 95% purity), with fractions containing the 8.8 kDa protein pooled together and concentrated to 20 mg/ml using a 3 kDa cut-off spin concentrator (Amicon Ultra-15).

### Size-exclusion chromatography coupled to multi-angle light scattering

SEC-MALS was carried out using an AKTA Pure25 (GE Healthcare) fitted with DAWN HELEOS-II 18 angle light-scattering detector and an Optilab T-rEX refractive index monitor (both Wyatt Technologies). SilF_38-120_ was applied to a Superdex 75 10/300 increase (GE Healthcare) pre-equilibrated with SEC buffer; 100 μl of sample was run at 2 mg/ml. Data was collected and analyzed using Astra v7 (Wyatt).

### Analytical ultracentrifugation

AUC sedimentation velocity experiments were carried out using the Beckman Optima analytical ultracentrifuge (Beckman). Samples were loaded into 12 mm double sector epoxy resin cells with sapphire windows at both ends, encased in an aluminum cell. Sample volumes were 400 μl reference SEC buffer and 396 μl solute. Concentrations of SilF_38-120_ used were as follows: 2.0 mg/ml, 1.0 mg/ml, 0.5 mg/ml, and 0.25 mg/ml. Ag(I) or Cu(I) was added to samples to a concentration of 10 mM for holo bound runs. The experiment was performed at 20 °C with a rotor speed of 50,000 rpm for 18 h to ensure complete sedimentation of protein. Radial scans were obtained using absorbance and Rayleigh interference optics with measurements made every 20 s and 120 s, respectively. Data was analyzed in SEDFIT((52)) using the continuous distribution (c(s)) method. Sedimentation coefficient and molecular mass are determined normalized to buffer density and viscosity at 20 °C. Buffer density and viscosity measurements were made using an Anton Paar DMA5000 with online viscometer.

### Isothermal titration calorimetry

ITC experiments were conducted using a MicroCal PEAQ-ITC calorimeter (Malvern Panalytical) in a Coy anaerobic chamber (<5 ppm O_2_). Purified SilF_38-120_ was dialyzed into water (for Ag(I) studies) or 1 M NaCl (for Cu(I) & Cu(II)) overnight, then diluted in each respective buffer to 25 μM before being injected into the sample cell. Metal titrants of 250 μM AgNO_3_ and 250 μM Cu(I)/Cu(II) were made in water and 1 M NaCl, respectively.

Injections of 1 μl metal titrant were spaced every 2 min for a total of 39 injections, with an initial injection of 0.4 μl; stirring of the cell was conducted at 750 rpm at a constant 25 °C. Data analysis was carried out using Microcal PEQA-ITC (https://www.malvernpanalytical.com/en/support/product-support/software/microcal-peaq-itc-family-analysis-software-update-v140) software (version 1.40, Malvern Panalytical).

### Crystallization and structure refinement

Purified SilF_38-120_ in SEC buffer was screened in several commercially available screening condition kits (SG1 and Morpheus (Molecular Dimensions)), using a protein concentration of 20 mg/ml. Screens of SilF_38-120_ were prepared both without (apo) and with (holo) Ag(I) (in the form of 5 mM AgNO_3_). Crystallization was carried out using the sitting drop method in CrystalQuick X plates (Grenier), with 1 nl drops mixed with 1 nl crystallization matrix left at 20 °C.

Crystals of apo- SilF_38-120_ formed within a couple of days in several conditions with the condition from the SG1 screen condition G3 (0.01 M ZnSO_4_, 0.1 M MES (pH 6.5), 25% v/v PEG 550 MME) opted for use. Further crystal optimization around this condition was conducted with crystals used from the final condition 0.01 M ZnSO_4_, 0.1 M MES (pH 7.6), 14% PEG 550 MME. Crystals were soaked in cryoprotectant for 30 s, then flash frozen in liquid nitrogen and stored.

Crystals of Ag(I)-SilF_38-120_ formed in SG1 screen D10 (0.2 M LiSO_4_, 0.1 M Bis-Tris pH 6.5, and 25% w/v PEG 3350) after approximately a week. Crystals were picked and soaked in cryoprotectant (supplemented with 27% PEG 3350, 15% glycerol, and 5 mM AgNO_3_) for 30 s before flash frozen in liquid nitrogen and stored. Crystals of Cu(I)-SilF_38-120_ also formed in the SG1 screen; however, this time condition C4 (0.2 M potassium sodium tartrate tetrahydrate and 20% w/v PEG 3350). Crystals grew after approximately 2 weeks in anaerobic conditions; the crystals were picked and soaked in cryoprotectant supplements with 27% PEG 3350, 15% glycerol, and 5 mM CuCl.

X-ray diffraction data was collected on beamline I24 at Diamond Light Source. The structures were solved by molecular replacement in Phaser (53). The apo structure was solved using CusF (PDB 2BV3) as a model; all subsequent structures were solved using the apo-SilF_38-120_ structure as the model. Further model building and refinement were carried out in Coot (54) and Refmac (55) (version 5.8.0258) respectively; refined models were evaluated through MolProbity (56). X-Ray and refinement data is given in Table S2.

### Hydrogen-deuterium exchange mass spectrometry

For initial peptide mapping, SilF_38-120_ (at 40 μM) was diluted ×11 in buffer E (20 mM Hepes, 30 mM KNO_3_, pH 7.8) and quenched 1:1 with 100 mM KH_2_PO_4_/K_2_HPO_4_, 2 M GuHCL, pH 2.08. Fifty microliters of this sample was injected into a Waters HDX Manager with an immobilized pepsin column (2.1 × 30 mm; Waters), C18 trapping column (VanGuard ACQUITY BEH 2.1 × 5 mm; Waters), and analytical C18 column (1.0 × 100 mm ACUITY BEH; Waters); mass spectrometer – Synapt G2-Si. Mobile phases were 0.1% formic acid in H_2_O (A) and 0.1% formic acid in ACN (B), such that their pH was 2.55. Protein was applied to the pepsin and trapping columns in A at 100 μl/min and eluted from the analytical column according to the following elution profile using H_2_O/ACN (+0.1% formic acid v/v): 1 to 7 min, 97% water to 65% water; 7 to 8 min, 65% water to 5% water, 8 to 10 min, held at 5% water.

Sample preparation of SilF_38-120_ in its apo and Ag(I)-bound states for labeling experiments were conducted in the same manner as mapping; however, sample buffer E was made in D_2_O instead of H_2_O (buffer L) and quenching occurred after 30 s, 5 min, and 30 min.

Peptide sequences were assigned from MSE fragment data with Protein Lynx Global Server 3.0.3 (Waters) and DynamX 3.0 (Waters). Labeling data was acquired as for sequencing, except the mass spectrometer–acquired MS scans only. Differences in uptake were filtered using hybrid significance testing using Deuteros 2.0 and overlaid on the SilF_38-120_ protein structure using Pymol.

### Circular dichroism

CD experiments were performed on beamline B23 of the Diamond Light Source using a nitrogen-flushed Chirascan Plus CD spectropolarimeter (Applied Photophysics Ltd). Five samples, all of 5 mg/ml concentration (and their corresponding buffers) were supplied: one native peptide, SilF apo, (buffer: Hepes 20 mM, KNO_3_ 30 mM), one peptide at pH 5 (buffer: CH_3_COONa 20 mM, KNO_3_ 30 mM), one at pH 9 (buffer: bicine 20 mM, KNO_3_ 30 mM), one peptide with Cu(I) ions (1:10 ratio, buffer: Hepes 20 mM, KNO_3_ 30 mM, 10 Cu(I) equivalent), and one peptide with Ag(I) ions (1:1 ratio, buffer: Hepes 20 mM, KNO_3_ 30 mM, 1 Ag(I) equivalent). The samples were studied across two regions: near-UV (250–330 nm) and far-UV (180–260 nm). The measurements were acquired using an integration time of 1 s, cuvettes of 0.002 cm (demountable – for far-UV), and 0.2 cm pathlength cuvette (for near-UV) with 1 nm bandwidth at 25 °C. Four repetitions were acquired for each sample. The data obtained was processed using CDApps—for the far-UV region (57) and OriginLab.

### Computational methods

#### Parametrization

Since the recent nonbonded set of classical parameters failed to correctly describe the metal coordination in the MD simulation of SilF_38-120_ protein, we generated bonded force field parameters for Cu(I) and Ag(I) ions using Seminario/ChgModB method available through the Python module of Metal Centre Parameter Builder (MCPB.py) in Amber18 (https://ambermd.org/doc12/Amber18.pdf) software. We used X-Ray structures of Cu(I)- SilF_38-120_ and Ag(I)- SilF_38-120_ which both have one histidine and two methionine residues in their metal coordination sites. We performed the geometry optimization and force constant calculations for the sidechain model and the Merz-Kollman RESP charge calculation for the large model using B3LYP/def2-TZVP level of theory in Gaussian16 program. The Lennard-Jones parameters for monovalent cations Cu(I) and Ag(I) were obtained from (58). Final force field parameters are available in the Supporting Information (SI) detailed above. We used the H++ webserver to determine the protonation states of the titratable residues in SilF_38-120_ protein using the physiological conditions (pH = 7, salinity = 0.15, internal dielectric = 10, external dielectric = 80). The ff14SB force field parameters were used to model the standard protein residues. The final systems were solvated in the truncated octahedron of TIP3P water molecules (10 Å from the solute) and neutralized adding chloride counterions. The apo form of SilF_38-120_ protein without a metal present was modeled at the physiological (pH = 7) conditions using a similar approach.

#### Classical MD simulations

Following the initial 1000 steps of solute-restrained (20 kcal mol^−1^) steepest descent minimization, we further relaxed the systems by performing Langevin MD simulations at the constant temperature (300 K with a collision frequency of 2 ps-1) and pressure (1 atm with a relaxation time of 2 ps using the Berendsen barostat) applying the equivalent weak positional harmonic restraints on protein atoms for a total of 1 ns. After a short relaxation, the systems were subjected to four parallel production *NPT* simulations (1.6 ms each) using 2 fs time step and SHAKE algorithm recording a snapshot every 2 ps. Periodic boundary conditions were applied in all directions, while Particle Mesh Ewald method with a cutoff of 12 Å was used to account for long-range electrostatics. All MD simulations were carried out using *pmemd* module, while the analysis of the resulting trajectories was performed using *cpptraj* tools of Amber18 software. Root mean square fluctuations for each residue *i* were calculated and converted to B-factors using the formula:

$$B_{i}=\left(\frac{8\pi^{2}}{3}\right){\left({RMSF}_{i}\right)}^{2}$$

#### QM/MM simulations

A set of previously obtained snapshots were further minimized for 300 and 200 steps of steepest descent and conjugated gradient unrestrained minimization respectively, using a coupled QM/MM potential. While a classical ff14SB force field has been chosen to describe MM atoms, the B3LYP/def2-TZVP method was used to treat QM atoms. The metal ion, the sidechains of two Met ,and one His residues were treated quantum mechanically. Minimized structures were subject to 2 ps of *NVT* equilibration before collecting the additional production simulation data for total of 40 ps recording a snapshot every 2 fs. A similar simulation setup has been employed as described earlier. All water molecules, counterions, and the rest of the protein was modeled classically. All QM/MM calculations were carried out using *sander* module of Amber18 program with the QM calculations performed externally employing Gaussian 16 package.

#### ONIOM calculations

To calculate the binding affinity, we extracted the suitable snapshots from QM/MM simulations and performed the two-layer ONIOM calculations using a full SilF protein in the presence and the absence of the metal (see Fig. 2). The residues found within 5 Å from the QM zone were allowed to move freely during the optimization, and nearest 200 water molecules around the QM region were retained. The metal and the sidechains of the coordinating residues were described with QM, while the rest of the protein, solvent, and counterions was treated using classical MM. The optimizations and frequency analysis were carried out using the ONIOM[B3LYP-D3/def2- TZVP:ff14SB] level of theory, employing the mechanical embedding followed by the electronic embedding single point calculation at the same level of theory. All ONIOM calculations were carried out with Gaussian16 (https://gaussian.com/gaussian16/) software. The solvation enthalpy of metal ions was obtained using the implicit SMD single point calculations.

## Data availability

PDB codes for deposited structures are Apo-SilF (8BBZ), Ag(I)-bound SilF (8BHU), and Cu(I)-bound SilF (8BWV). All simulation files are freely available online at https://doi.org/10.6084/m9.figshare.21285264.v1. The data consists of (a) Molecular dynamics and QM/MM input, parameter, coordinates, and parameter files, (b) Force field parameterization files, (c) run-scripts for setup and production runs, (d) coordinate output files for QM/MM calculations, (e) analysis scripts and results.

## Supporting information

This article contains supporting information.

## Conflict of interest

The authors declare that they have no conflicts of interest with the contents of this article.
