# Supplementary material for: The adaptability of the ion-binding site by the Ag(I)/Cu(I) periplasmic chaperone SilF
Source: J Biol Chem. 2023 Oct 14;299(11):105331. doi: 10.1016/j.jbc.2023.105331 (PMC10656224; doi:10.1016/j.jbc.2023.105331)
Supplement: Supporting Information [file mmc1.docx]

SUPPLEMENTARY INFORMATION

The adaptability of ion binding site by the Ag(I)/Cu(I) periplasmic chaperone SilF.

Ryan M. Lithgo^1,2,3,4,9^, Marko Hanževački^598^, Gemma Harris^4^, Jos J. A. G. Kamps^3,4^, Ellie Holden^6^, Tiberiu-Marius Gianga^3^, Justin LP Benesch^6^, Christof M. Jäger^5,7^, Anna K. Croft^8^, Rohannah Hussain^3^, Jon L. Hobman^1^, Allen M. Orville^3,4^, Andrew Quigley^2,3,4^, Stephen B. Carr^4,6^, David J. Scott^1,4,10^

^1^School of Biosciences, University of Nottingham, Sutton Bonington Campus, Leicestershire, LE12 5RD, United Kingdom.

^2^Membrane Protein Laboratory, Diamond Light Source, Rutherford Appleton Laboratory, Didcot, Oxfordshire, OX11 0FA, United Kingdom.

^3^Diamond Light Source, Diamond House, Rutherford Appleton Laboratories, Didcot, Oxfordshire, OX11 0FA, United Kingdom.

^4^Research Complex at Harwell, Rutherford Appleton Laboratory, Didcot, Oxfordshire, OX11 0FA, United Kingdom.

^5^Department of Chemical and Environmental Engineering, University of Nottingham, University Park, Nottingham NG7 2RD. United Kingdom.

^6^Department of Chemistry, University of Oxford, Oxford, Oxfordshire, OX1 3QU, United Kingdom.

^7^Data Science and Modelling, Pharmaceutical Sciences, R&D, AstraZeneca Gothenburg, Pepparedsleden 1, SE-431 83 Mölndal, Sweden.

8Department of Chemical Engineering, University of Loughborough, Loughborough LE11 3TU, United Kingdom

^9^Equal contribution

^10^Corresponding Author

**SUPPLEMENTARY FILES**

**Protein Databank Codes**

- Apo-SilF: 8BBZ
- Ag(I) bound SilF: 8BHU
- Cu(I) bound SilF: 8BWV

**Simulation Files**

All simulation files are freely available online at:

<https://doi.org/10.6084/m9.figshare.21285264.v1>

data consists of:

Computational supporting data consisting of:

- Molecular dynamics and QM/MM input, parameter, coordinates, and parameter files
- Force field parameterisation files
- run-scripts for setup and production runs
- coordinate output files for QM/MM calculations
- analysis scripts and results

**SUPPLEMENTARY TABLES**

**Supplementary Table 1: SEC-MALS analysis of SilF_38-120_**

| SEC-MALS Output | Values |
| --- | --- |
| Radius of hydration (rh(Q)z) (nm) | 2.323 (±8.365%) |
| Average rh(Q) (nm) | 1.884 (±1.670%) |
| Number averaged molecular weight (Mn) (g/mol) | 8.743x10^3^ (±7.888%) |
| Mp (g/mol) | 8.664x10^3^ (±7.082%) |
| Weight averaged molecular weight (Mw) (g/mol) | 8.769x10^3^ (±8.116%) |
| Polydispersity (Mw/Mn) | 1.003 (±4.398%) |

**Supplementary Table 2: Protein crystallography refinement statistics**

|  | apo-SilF | Ag(I)-SilF | Cu(I)-SilF |
| --- | --- | --- | --- |
| Wavelength | 0.999 Å | 0.999 Å | 0.999 Å |
| Resolution range | 45.95 - 2.2 (2.279 - 2.2) | 51.41 - 1.7 (1.761 - 1.7) | 46.92 - 2.2 (2.279 - 2.2) |
| Space group | P 65 2 2 | P 21 21 21 | I 21 21 21 |
| Unit cell | 109.47 109.47 84.59,  90 90 120 | 60.93 81.59 95.78,  90 90 90 | 77.29 77.29 187.69,  90 90 90 |
| Total reflections | 572835 (46562) | 713161 (65325) | 701243 (47107) |
| Unique reflections | 15524 (1502) | 53221 (5226) | 28977 (2855) |
| Multiplicity | 36.9 (31.0) | 13.4 (12.5) | 24.2 (16.5) |
| Completeness (%) | 98.87 (97.85) | 99.95 (100.00) | 99.90 (99.40) |
| Mean I/sigma(I) | 15.1 (1.0) | 12.8 (0.7) | 7.8 (1.8) |
| Wilson B-factor | 46.35 | 26.62 | 43.13 |
| CC1/2 | 1.0 (0.6) | 0.998 (0.328) | 0.997 (0.901) |
| Reflections used in refinement | 15521 (1502) | 53213 (5226) | 28904 (2852) |
| Reflections used for R-free | 802 (74) | 2618 (274) | 1420 (150) |
| R-work | 0.2154 (0.2819) | 0.2025 (0.2823) | 0.2794 (0.3935) |
| R-free | 0.2493 (0.3449) | 0.2335 (0.2584) | 0.3197 (0.4211) |
| Number of non-hydrogen atoms | 1805 | 3790 | 3655 |
| macromolecules | 1792 | 3563 | 3634 |
| ligands | 2 | 60 | 12 |
| solvent | 11 | 167 | 9 |
| Protein residues | 235 | 469 | 478 |
| RMS(bonds) | 0.014 | 0.015 | 0.010 |
| RMS(angles) | 1.94 | 1.84 | 1.21 |
| Ramachandran favored (%) | 98.69 | 99.78 | 94.21 |
| Ramachandran allowed (%) | 1.31 | 0.22 | 5.58 |
| Ramachandran outliers (%) | 0.00 | 0.00 | 0.21 |
| Rotamer outliers (%) | 2.03 | 0.00 | 1.75 |
| Molprobity score | 1.50 | 1.21 | 2.40 |
| Average B-factor | 53.13 | 33.59 | 53.66 |

**Supplementary Table S3: Bond lengths for Ag(I) and Cu(I) bound to SilF and CusF, respectively.**

| **Bond**  **(X = metal ion)** | **SilF-Ag(I) (Å)** | **CusF- Ag(I) (Å)** | **SilF- Cu(I) (Å)** | **CusF- Cu(I) (Å)** | **SilF-Ag(I)**  **QM/MM^1^** | **SilF-Cu(I)**  **QM/MM^1^** | **SilF-Ag(I)**  **Classical** | **SilF-Cu(I)**  **Classical** |
| --- | --- | --- | --- | --- | --- | --- | --- | --- |
| N-X (His60) | 2.3 | 2.2 | 2.3 | 2.0 | 2.3 | 2.1 | 2.3 | 2.0 |
| S-X (Met73) | 2.7 | 2.7 | 2.2 | 2.2 | 2.8 | 2.4 | 2.6 | 2.3 |
| S-X (Met75) | 2.5 | 2.4 | 2.4 | 2.2 | 2.6 | 2.4 | 2.6 | 2.3 |
| CE3/CZ3-X (Trp70) | 2.8/2.9 | 2.8/3.0 | - | 2.7/2.9 | 3.7/4.0 | 4.0/4.3 | 3.3/3.7 | 3.8/4.2 |
| H_2_O-X | - | - | 2.5 |  | 6.6 | 1.8 | 2.7 | 2.1 |

**SUPPLEMENTARY FIGURES**

**Supplementary Figure 1**


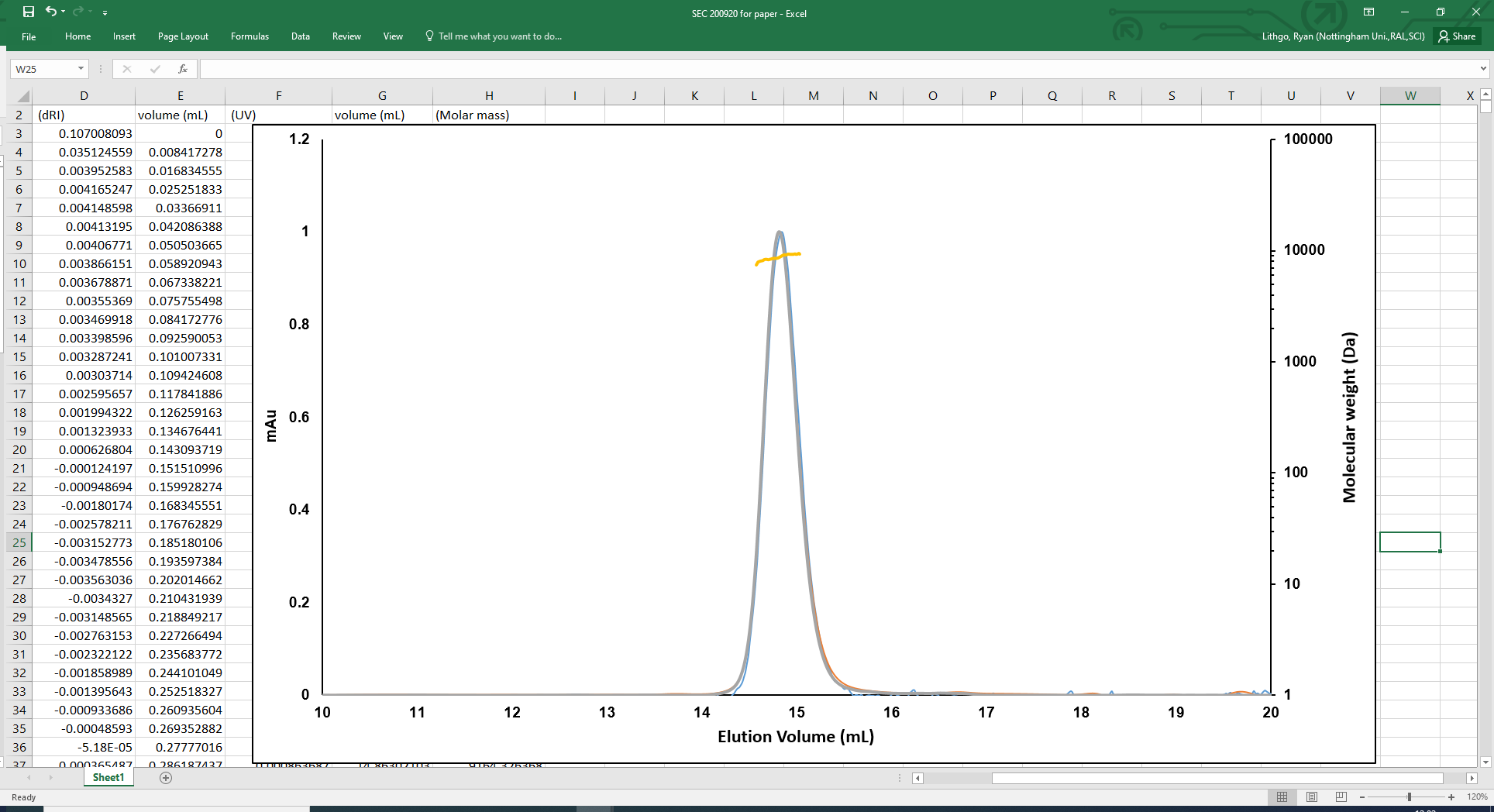


**Figure S1:** SEC-MALS analysis of SilF. The chaperone elutes as a single peak as judged by the UV absorbance trace taken at 280 nm. The calculated molecular weight across the peak is shown in yellow and corresponds to that expected for a monomer of 9 kDa.

**Supplementary Figure 2:**

**
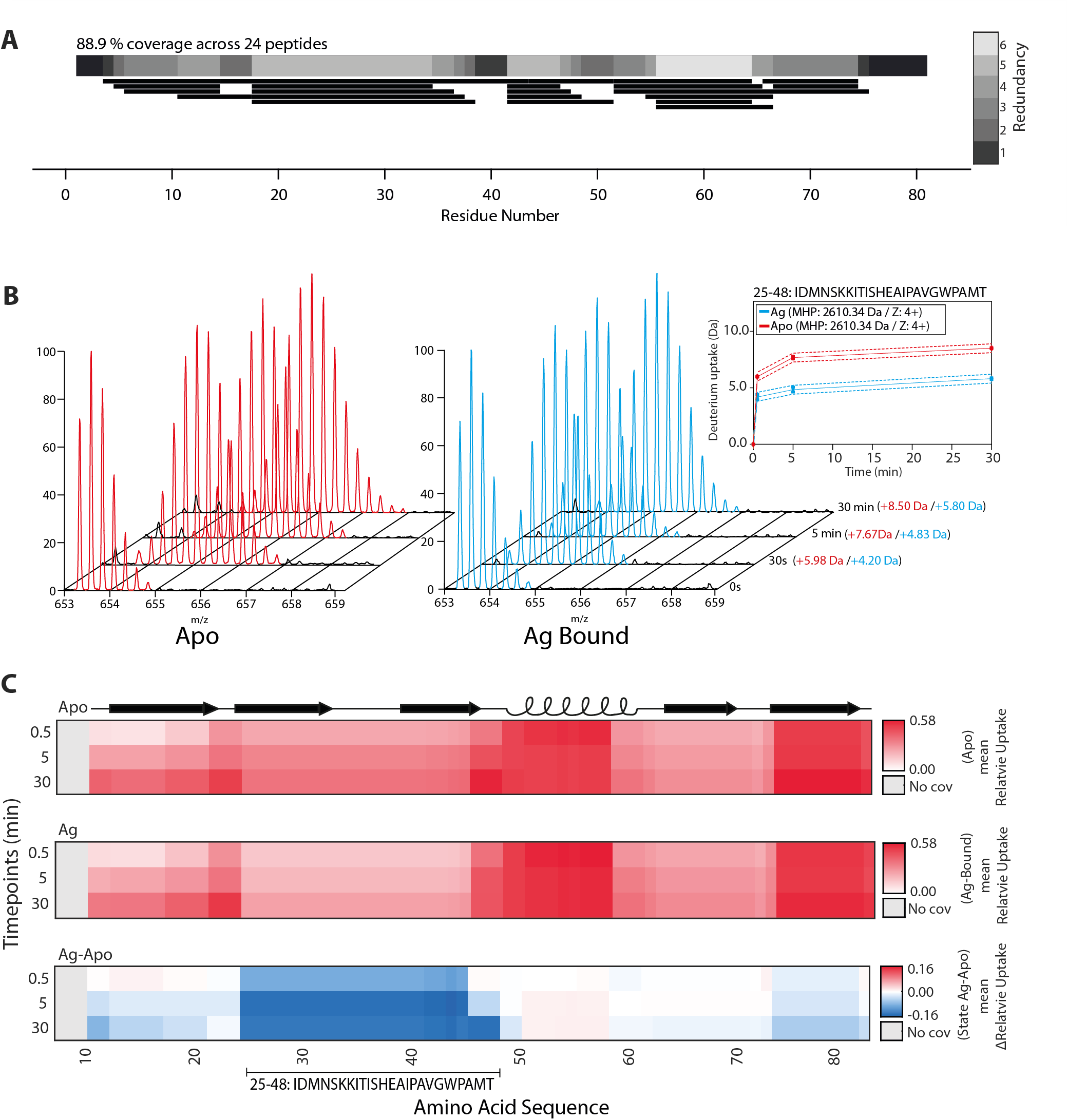
**

**Figure S2: A** shows a coverage map of the peptic peptides identified along the amino acid sequence of SilF. **B** is the raw spectra of peptide IDMNSKKITISHEAIPAVGWPAMT (residues 52-75) and a combined deuterium uptake plot over time. The apo state results in a far greater m/z shift over all timepoints, than Ag(I) bound, due to the protection of the amino acids interacting with Ag(I) when in the bound state. **C** illustrates this difference in the context of the whole amino acid sequence in the form of a deuterated heatmap, for the apo state, the Ag-bound state and the difference between the states. The major difference is localized to beta-strand 2&3 as well as the linking loop between them.
